# Resveratrol in diabetes and pancreatic function: implications for the exocrine–endocrine pancreatic axis–a systematic review

**DOI:** 10.3389/fnut.2026.1806881

**Published:** 2026-04-22

**Authors:** Ana Meden, Dušanka Mičetić-Turk

**Affiliations:** 1 University Medical Centre Maribor, Maribor, Slovenia; 2 Faculty of Medicine, University of Maribor, Maribor, Slovenia

**Keywords:** diabetes mellitus, exocrine–endocrine pancreatic axis, inflammation, oxidative stress, pancreatic function, pancreatogenic diabetes, resveratrol, β-cell protection

## Abstract

Pancreatic dysfunction plays an important role in the development and progression of diabetes mellitus. Resveratrol, a naturally occurring polyphenolic compound, has attracted considerable interest due to its antioxidant, anti-inflammatory, and metabolic regulatory properties. The aim of this systematic review was to synthesize available evidence regarding the metabolic and pancreatic effects of resveratrol in diabetes mellitus. A systematic literature search of the PubMed/MEDLINE database (2011–2025) identified preclinical studies, randomized controlled trials, systematic reviews, and meta-analyses investigating resveratrol in diabetic contexts. Evidence from experimental and clinical studies indicates that resveratrol may improve glycemic control, attenuate inflammatory responses, reduce oxidative stress, and protect pancreatic β-cell function, primarily through activation of signaling pathways such as sirtuin-1 (SIRT1) and AMP-activated protein kinase (AMPK). Most available evidence originates from studies in type 2 diabetes mellitus or experimental models of diabetes. Nevertheless, several mechanisms identified in these studies–including modulation of oxidative stress, inflammation, and pancreatic tissue remodeling–may also be relevant to diseases characterized by combined exocrine and endocrine pancreatic dysfunction. In this context, resveratrol may influence biological processes related to the exocrine–endocrine pancreatic axis, including pancreatic inflammation, fibrosis, and β-cell preservation. Overall, current evidence supports the metabolic and pancreatic protective effects of resveratrol in diabetes. However, direct studies investigating its role in pancreatogenic diabetes remain scarce. Future research specifically targeting pancreatic diseases associated with diabetes is needed to clarify the therapeutic potential of resveratrol in disorders affecting the exocrine–endocrine pancreatic axis.

## 1 Introduction

Pancreatogenic diabetes mellitus (PDM), also referred to as type 3c diabetes, is a form of diabetes resulting from diseases of the exocrine pancreas. It commonly develops in the context of chronic pancreatitis, pancreatic cancer, cystic fibrosis, or following pancreatic surgery. Unlike type 1 and type 2 diabetes, PDM is characterized by combined endocrine and exocrine pancreatic dysfunction. Loss of pancreatic parenchyma, fibrosis, and inflammation impair insulin secretion and may also lead to deficiencies in glucagon and pancreatic polypeptide. In addition, exocrine insufficiency contributes to nutrient malabsorption and altered incretin signaling, which further complicate glycemic regulation. Although increasingly recognized, PDM remains underdiagnosed and often inadequately managed (1, 2).

Resveratrol is a naturally occurring polyphenolic compound found in various plants, including grapes, berries, and peanuts. It has attracted considerable attention due to its antioxidant, anti-inflammatory, cardioprotective, and metabolic regulatory properties (3, 4). Experimental studies suggest that resveratrol modulates several cellular signaling pathways, including activation of sirtuin-1 (SIRT1) and AMP-activated protein kinase (AMPK), which are involved in metabolic homeostasis, mitochondrial function, and cellular stress responses. Through these mechanisms, resveratrol has been investigated as a potential therapeutic agent in metabolic disorders, including diabetes mellitus (3–5).

A growing body of literature has examined the effects of resveratrol on glucose metabolism, insulin sensitivity, oxidative stress, and inflammatory signaling in both experimental models and clinical studies of diabetes, particularly type 2 diabetes mellitus (2, 6–8). These studies indicate that resveratrol may improve glycemic control, enhance antioxidant defense mechanisms, and protect pancreatic β-cell function. However, most available evidence originates from studies focusing on metabolic forms of diabetes rather than pancreatic disease-related diabetes (6, 9–12).

Given the importance of the exocrine–endocrine pancreatic axis in the development of pancreatogenic diabetes, biological mechanisms influencing pancreatic inflammation, oxidative stress, and tissue regeneration may be of particular interest (3, 13). Compounds such as resveratrol, which target these pathways, could potentially influence pancreatic function beyond their systemic metabolic effects.

The aim of this systematic review is therefore to synthesize available evidence on the metabolic and pancreatic effects of resveratrol in diabetes mellitus. Particular attention is given to mechanisms relevant to pancreatic function, including oxidative stress, inflammation, and β-cell protection. Based on these findings, the review also discusses potential implications for the exocrine–endocrine pancreatic axis and the pathophysiology of pancreatogenic diabetes.

## 2 Methods

This systematic review was conducted in accordance with the Preferred Reporting Items for Systematic Reviews and Meta-Analyses (PRISMA) guidelines.

### 2.1 Research question

The research question was formulated using the PICOS framework:

1. Population (P): humans with diabetes mellitus or experimental models of diabetes and pancreatic dysfunction
2. Intervention (I): resveratrol supplementation or experimental exposure to resveratrol
3. Comparison (C): placebo, no treatment, or standard care
4. Outcomes (O): effects on glucose metabolism, insulin sensitivity, oxidative stress, inflammatory markers, pancreatic β-cell function, and pancreatic tissue changes
5. Study design (S): randomized controlled trials, systematic reviews, meta-analyses, and relevant preclinical studies.

The review aimed to identify evidence regarding the metabolic and pancreatic effects of resveratrol in diabetes and to explore potential implications for pancreatic function and the exocrine–endocrine pancreatic axis.

### 2.2 Search strategy

A systematic literature search was performed in the PubMed/MEDLINE database for studies published between January 2011 and December 2025. The search strategy combined Medical Subject Headings (MeSH) and free-text terms related to resveratrol and diabetes.

The following search terms were used:

(“resveratrol”) AND (“diabetes mellitus” OR “type 2 diabetes” OR “pancreatogenic diabetes” OR “type 3c diabetes” OR “pancreatic function” OR “exocrine pancreas” OR “endocrine pancreas” OR “oxidative stress” OR “inflammation” OR “pancreatic regeneration”)

Boolean operators (AND/OR) were applied to combine search terms. Filters were used to include English-language publications and studies involving humans or experimental models.

Reference lists of eligible studies were also manually screened to identify additional relevant publications.

### 2.3 Study selection, data extraction, and analysis

Titles and abstracts were initially screened by the first author (AM). Full texts of potentially relevant studies were subsequently assessed for eligibility. The second author (DM-T) independently reviewed the selected articles. Any discrepancies were resolved through discussion and consensus.

For each included study, the following data were extracted:

1. Study design
2. Study population or experimental model
3. Intervention characteristics (dose and duration of resveratrol exposure)
4. Main outcomes related to metabolic parameters and pancreatic function.

The eligibility criteria used for study selection were defined according to the PICOS framework and are summarized in Table 1.

**TABLE 1:** Eligibility criteria for study selection based on the PICOS framework.

Component	Inclusion criteria	Exclusion criteria
Population (P)	Humans with diabetes mellitus (primarily type 2 diabetes) or experimental models of diabetes and pancreatic dysfunction (animal or *in vitro* studies)	Studies focusing on non-diabetic populations without metabolic or pancreatic relevance
Intervention (I)	Resveratrol supplementation or experimental exposure to resveratrol	Studies not evaluating resveratrol or investigating other polyphenols without resveratrol-specific outcomes
Comparison (C)	Placebo, no treatment, standard care, or control group in experimental studies	Studies lacking a comparator or control condition
Outcomes (O)	Outcomes related to glucose metabolism, insulin sensitivity, oxidative stress, inflammation, pancreatic β-cell function, pancreatic regeneration, or pancreatic tissue changes	Studies not reporting metabolic or pancreatic-related outcomes
Study design (S)	Randomized controlled trials, systematic reviews, meta-analyses, narrative reviews, and relevant preclinical studies	Case reports, editorials, conference abstracts, letters to the editor, and studies with insufficient methodological detail
Publication characteristics	Articles published between 2011 and 2025, written in English, available in full text	Non-English publications, studies published outside the defined time frame, or articles without accessible full text

These criteria were applied during both the title/abstract screening and the full-text review stages.

### 2.4 Data synthesis

Due to heterogeneity in study designs, populations, and reported outcomes, a quantitative meta-analysis was not performed. Instead, a qualitative narrative synthesis was conducted. Studies were grouped into the following categories:

1. Preclinical studies (animal and *in vitro* models)
2. Clinical studies (randomized controlled trials and meta-analyses)
3. General reviews summarizing mechanisms and clinical evidence

### 2.5 Risk of bias assessment

Due to the heterogeneity of included study designs, risk of bias was assessed narratively rather than using a single standardized assessment tool. For randomized controlled trials, aspects such as randomization procedures, allocation concealment, blinding, and completeness of outcome reporting were considered. For preclinical studies, potential sources of bias included lack of blinding, small sample sizes, and variability in experimental protocols. Reviews and meta-analyses were evaluated with respect to methodological transparency and potential publication bias.

## 3 Results

### 3.1 PRISMA flow diagram

**FIGURE 1:**
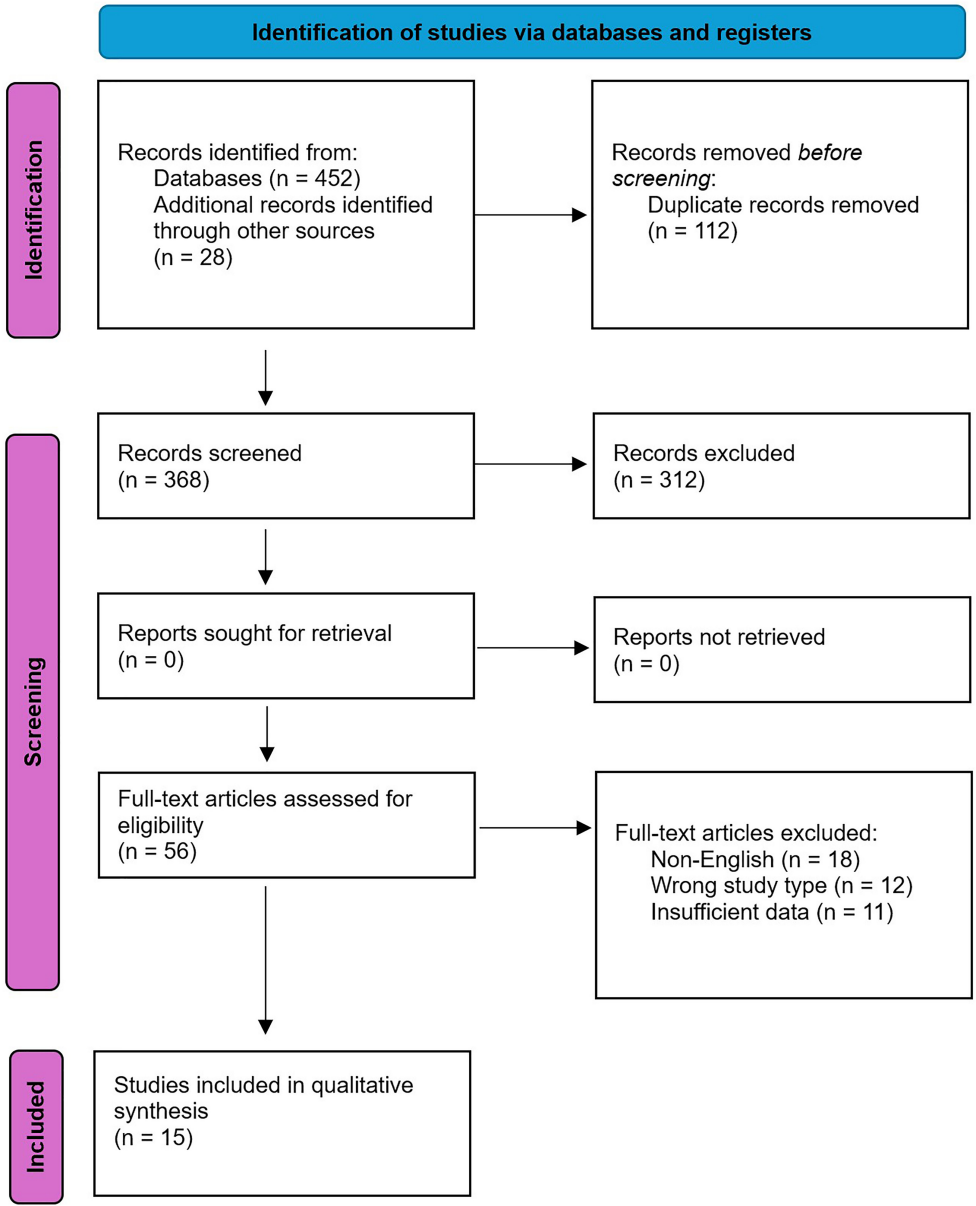
Summary of PRISMA flow diagram.

The study selection process is illustrated in the PRISMA analysis table (Figure 1, adapted from PRISMA guidelines). The initial database search yielded 452 records, with an additional 28 identified through reference lists (total 480). After removing duplicates (*n* = 112), 368 records remained for title and abstract screening. Of these, 312 were excluded (e.g., irrelevant topics, non-diabetes focus). Full-text assessment of 56 articles led to the exclusion of 41 (e.g., 18 non-English, 12 wrong study type, 11 insufficient data). Ultimately, 15 studies were included in the qualitative synthesis.

### 3.2 Preclinical studies

These studies focus on animal models (e.g., rats, mice), *in vitro* experiments, or reviews emphasizing preclinical mechanisms in diabetes models with implications for pancreatic function (Table 2).

**TABLE 2:** Summary of preclinical (animal and *in vitro*) studies.

Study	Design	Participants/model	Intervention/dose/duration	Key outcomes
Ciddi and Dodda (11)	*In vitro*/*in vivo* review	Diabetic rats/cells	Variable	Therapeutic potential in complications; inhibited polyol pathway, protected against gluco/lipotoxicity
Chen et al. (21)	Preclinical study	STZ-induced DM rats	Resveratrol + ADSC transplantation	Enhanced regeneration, activated AMPK/SIRT1, reduced apoptosis/fibrosis
Cheng et al. (27)	Preclinical study	MG-induced DM mice	Oral resveratrol	Improved glucose tolerance, increased insulin content, Nrf2 activation
Chen et al. (22)	Preclinical study	STZ-induced DM rats	Resveratrol-preconditioned ADSC	Increased stem cell viability (p-Akt), better glucose control, improved histology
Lee et al. (20)	Preclinical study	NOD mice (T1D)	Oral/injected resveratrol	Reduced CCR6, prevented insulitis, reversed diabetes
Vetterli et al. (26)	*In vitro* study	INS-1E cells/human islets	Resveratrol exposure	Potentiated GSIS via SIRT1; upregulated beta-cell genes
Koushki et al. (12)	Critical review	DM models	N/A	Prevention/therapy of complications; anti-apoptotic, anti-fibrotic effects in pancreas
Hoca et al. (5)	Narrative review	DM/obesity models	N/A	Role in insulin resistance; modulated adipokines, reduced obesity-associated DM burden (primarily model-based)

### 3.3 Human/clinical studies

These studies involve human participants (e.g., T2DM patients) in RCTs or meta-analyses/reviews summarizing clinical data, with implications for diabetes management (Table 3).

**TABLE 3:** Summary of human/clinical studies (RCTs, meta-analyses, and related reviews).

Study	Design	Participants/model	Intervention/dose/duration	Key outcomes
Mahjabeen et al. (7)	RCT	66 T2DM patients	1 g/day, 45 days	Reduced FPG (17.1%), HbA1c (0.5%), insulin (18.6%), oxidative stress (MDA), inflammation (TNF-α, hs-CRP)
Gu et al. (2)	Systematic review/meta-analysis	T2DM patients (11 RCTs, *n* = 388)	Variable doses, 4–24 weeks	MD: FPG −19.7 mg/dL, HbA1c −0.43%, HOMA-IR −0.88; greater benefits >500 mg/day, >3 months
Jeyaraman et al. (4)	Cochrane systematic review	T2DM patients (9 RCTs, *n* = 362)	Variable, 4–52 weeks	Low-certainty HbA1c reduction (MD −0.34%); inconsistent FPG/insulin; minimal adverse events

### 3.4 Mixed/general reviews

These are broader narrative or mini-reviews that span both preclinical and clinical aspects or general populations (Table 4).

**TABLE 4:** Summary of mixed/general reviews (not strictly preclinical or clinical).

Study	Design	Participants/model	Intervention/dose/duration	Key outcomes
Cory et al. (15)	Mini-review	General population	N/A	Role of polyphenols in health; mixed effects of resveratrol on metabolic parameters
Ahmadzadeh et al. (3)	Narrative review	DM models/patients	N/A	Mechanisms: SIRT1/AMPK activation, beta-cell protection, gut microbiota modulation, anti-inflammatory effects via NF-κB/NLRP3
Nanjan and Betz (10)	Narrative review	DM models/patients	N/A	Management of diabetes pathologies; beta-cell preservation, reduced AGEs/RAGE signaling
Szkudelski and Szkudelska (13)	Narrative review	Animal/human DM studies	Variable	From animal to human: improved homeostasis, beta-cell protection, AMPK/SIRT1 activation

### 3.5 Synthesized findings

#### 3.5.1 Clinical evidence on resveratrol in diabetes management

Clinical studies have demonstrated resveratrol’s efficacy in improving metabolic profiles in type 2 diabetes mellitus (T2DM), a condition sharing pathophysiological overlaps with PDM, such as insulin resistance and chronic inflammation (2, 4–6). In a randomized, placebo-controlled trial involving 66 T2DM patients, resveratrol supplementation at 1 g/day for 45 days significantly lowered fasting blood glucose (by 17.1%), HbA1c (by 0.5%), and insulin levels (by 18.6%), alongside reductions in oxidative stress markers like MDA and inflammatory cytokines (7). Expanded analyses from meta-studies confirm these trends, with one review of 17 RCTs showing dose-dependent improvements, particularly at >300 mg/day (8).

A systematic review and meta-analysis of 11 randomized controlled trials (RCTs) encompassing 388 T2DM participants confirmed these benefits, reporting mean differences of −19.7 mg/dL in fasting plasma glucose, −0.43% in HbA1c, and −0.88 in homeostasis model assessment of insulin resistance (HOMA-IR) (2). Subgroup analyses indicated enhanced effects with doses exceeding 500 mg/day and treatment durations over 3 months. Similarly, a Cochrane review of 9 RCTs (*n* = 362) found low-certainty evidence for HbA1c reduction (−0.34%), though effects on fasting glucose and insulin were inconsistent, possibly due to heterogeneity in study designs (4). Another meta-analysis highlighted resveratrol’s broader metabolic benefits, including improved lipid profiles and liver enzymes in patients with metabolic syndrome, which often co-occurs with diabetes (9).

Narrative reviews further elucidate resveratrol’s role in preventing diabetic complications. It attenuates downstream pathologies like neuropathy, nephropathy, and retinopathy by inhibiting advanced glycation end-products (AGEs) formation and receptor for AGEs (RAGE) signaling (5, 10, 11).

*In vivo* studies in streptozotocin-induced diabetic rats showed resveratrol restoring beta-cell mass and function, with *in vitro* evidence supporting protection against glucotoxicity and lipotoxicity (11, 12). Human trials also suggest resveratrol improves glycemic control and decreases insulin resistance in type 2 diabetic patients, supporting its potential as an adjunct therapy (13). In contexts relevant to PDM, such as pancreatic cancer-associated diabetes, resveratrol’s activation of SIRT1 may mitigate glucose metabolism disruptions (14).

While polyphenols like resveratrol show mixed results in broader health contexts, such as lipid metabolism, their consistent antidiabetic effects underscore therapeutic potential (15). A recent comprehensive review highlighted mechanisms including gut microbiota modulation, which could indirectly benefit exocrine pancreatic function by reducing systemic inflammation from dysbiosis (3). In obesity-associated diabetes, resveratrol’s impact on adipokines may further alleviate insulin resistance, indirectly supporting pancreatic function (5).

Additional evidence from high-glucose models demonstrates resveratrol’s attenuation of inflammation and endothelial dysfunction, expanding its applicability to vascular complications in diabetes (16).

#### 3.5.2 Implications for the exocrine-endocrine pancreatic axis and pancreatogenic diabetes

Although direct investigations in PDM are scarce, resveratrol’s biological actions may involve pathways relevant to the exocrine–endocrine pancreatic axis. In PDM, exocrine insufficiency leads to nutrient malabsorption, amplifying oxidative stress and inflammation that impair endocrine function (17). Resveratrol’s ability to protect acinar cells from oxidative damage–via Nrf2 activation–and inhibit inflammatory cascades could preserve exocrine secretion and prevent secondary beta-cell loss (13).

For instance, by modulating high-mobility group box 1 (HMGB1) and toll-like receptor 4 (TLR4), experimental evidence suggests that resveratrol may modulate inflammatory pathways involved in pancreatitis-associated pancreatic injury (18). Animal models demonstrate resveratrol reducing pancreatic fibrosis and apoptosis, maintaining islet architecture (19). In autoimmune models, it blocks migration of pro-inflammatory cells to the pancreas, potentially mitigating the autoimmune destruction seen in some PDM cases post-pancreatitis (20). Furthermore, resveratrol’s enhancement of stem cell-based regeneration could aid in repairing exocrine-endocrine damage following pancreatic injury (21, 22).

In human contexts, its insulin-sensitizing effects observed in diabetes studies may theoretically contribute to improved metabolic control in conditions involving pancreatic dysfunction (23). Resveratrol’s capability to improve insulin secretion and protect beta-cells, as seen in both animal and preliminary human studies, further supports its role in maintaining the pancreatic axis (13, 20).

Challenges include resveratrol’s low bioavailability, rapid metabolism to glucuronides and sulfates, necessitating optimized formulations like micronized or liposomal versions (24). Safety profiles from trials indicate minimal adverse effects, primarily mild gastrointestinal symptoms, with no significant hepatotoxicity or nephrotoxicity (25).

### 3.6 Risk of bias in included studies

Due to the heterogeneity of included study designs, risk of bias was assessed narratively rather than through a formal quantitative tool. For preclinical studies (e.g., animal models), biases included lack of blinding in some experiments and variability in dosing, potentially overestimating effects (6, 8–12, 14–16). Clinical RCTs generally had low risk for randomization and allocation concealment but moderate risk for blinding and small sample sizes [e.g., *n* = 66 in (17)], leading to potential performance and detection biases (2, 17, 18, 20–22).

Reviews and meta-analyses had low to moderate risk, with issues like publication bias and heterogeneity noted in Cochrane assessments (18). Overall, evidence quality is moderate, with stronger consistency in preclinical mechanisms but limited generalizability to human PDM.

## 4 Discussion

This systematic review synthesizes available evidence on the metabolic and pancreatic effects of resveratrol in diabetes mellitus and explores their potential relevance for pancreatic dysfunction. Overall, findings from preclinical studies, clinical trials, and meta-analyses suggest that resveratrol exerts multiple biological effects relevant to glucose metabolism, oxidative stress, inflammation, and pancreatic β-cell function (2, 3, 6, 13). These mechanisms have been widely investigated in type 2 diabetes mellitus (T2DM), where resveratrol has demonstrated improvements in glycemic control and insulin sensitivity in both experimental models and human studies (2, 17, 21).

### 4.1 Differences between type 2 diabetes and pancreatogenic diabetes

Although most studies included in this review focused on T2DM, pancreatogenic diabetes mellitus (PDM) represents a distinct clinical entity with different pathophysiological mechanisms. T2DM is primarily characterized by peripheral insulin resistance and progressive β-cell dysfunction, often associated with obesity and chronic low-grade inflammation. In contrast, PDM develops as a consequence of structural pancreatic damage caused by chronic pancreatitis, pancreatic cancer, cystic fibrosis, or pancreatic surgery (1, 25).

In PDM, the loss of pancreatic parenchyma, fibrosis, and chronic inflammation impair both endocrine and exocrine pancreatic function. Patients frequently present with deficiencies of insulin, glucagon, and pancreatic polypeptide, resulting in unstable glycemic control. Additionally, exocrine pancreatic insufficiency contributes to malabsorption and altered incretin signaling, further complicating metabolic regulation (1, 25). Because of these differences, therapeutic approaches developed for T2DM cannot be directly extrapolated to PDM.

### 4.2 Mechanisms of resveratrol relevant to pancreatic function

Despite the limited direct evidence in PDM, several mechanisms described in diabetes research may be relevant to pancreatic injury and repair. Experimental studies indicate that resveratrol exerts antioxidant and anti-inflammatory effects through modulation of signaling pathways such as SIRT1 and AMPK, which are involved in metabolic regulation and cellular stress responses (3, 6, 13). *In vitro* studies have demonstrated that resveratrol enhances glucose-stimulated insulin secretion and improves β-cell function through SIRT1-dependent mechanisms (12).

Animal studies further suggest protective effects on pancreatic tissue. For example, resveratrol has been shown to reduce oxidative stress, improve glucose tolerance, and increase pancreatic insulin content in experimental diabetes models (10). Additional studies report that resveratrol enhances pancreatic regeneration and reduces fibrosis and apoptosis when combined with stem cell therapy in diabetic rats (8, 9). These findings suggest that resveratrol may influence biological processes involved in pancreatic inflammation, cellular survival, and tissue repair.

### 4.3 Evidence from clinical studies

Clinical evidence regarding resveratrol supplementation in diabetes primarily derives from studies in patients with T2DM. Randomized controlled trials have reported improvements in fasting plasma glucose, HbA1c, insulin resistance, and inflammatory markers following resveratrol supplementation (17). Meta-analyses of randomized trials similarly demonstrate modest improvements in glycemic control, particularly at higher doses and longer treatment durations (2, 21).

However, results across studies are not entirely consistent. A Cochrane review evaluating resveratrol supplementation in adults with T2DM reported only low-certainty evidence for reductions in HbA1c and inconsistent effects on other glycemic parameters (18). These discrepancies likely reflect heterogeneity in study design, dosing regimens, treatment duration, and patient characteristics.

Beyond glycemic control, resveratrol has also been investigated for its potential role in preventing diabetes-related complications. Experimental and clinical studies suggest beneficial effects on oxidative stress, inflammation, and endothelial function, which may contribute to protection against microvascular and macrovascular complications (3, 14, 24).

### 4.4 Potential implications for the exocrine–endocrine pancreatic axis

Although direct clinical studies in PDM are lacking, several mechanisms identified in experimental diabetes research may be relevant to the exocrine–endocrine pancreatic axis. Oxidative stress and inflammation play central roles in pancreatic injury and fibrosis associated with chronic pancreatitis and other pancreatic diseases (3, 6). Through its antioxidant and anti-inflammatory properties, resveratrol may influence these processes and potentially modulate pancreatic tissue damage.

Experimental studies also suggest that resveratrol may reduce pancreatic fibrosis and inflammatory signaling pathways associated with pancreatic injury (26). Furthermore, its ability to enhance stem cell-mediated regeneration and protect β-cell function could theoretically contribute to preservation of pancreatic endocrine capacity following pancreatic damage (8, 9, 12). Nevertheless, these observations are largely derived from experimental models and should therefore be interpreted cautiously.

### 4.5 Limitations and future directions

Several limitations should be considered when interpreting the findings of this review. First, the majority of available studies investigate resveratrol in the context of T2DM or experimental models of diabetes rather than pancreatogenic diabetes. Consequently, the implications for PDM remain largely hypothetical. Second, many clinical trials involve relatively small sample sizes and heterogeneous study designs, which may contribute to inconsistent results across studies (2, 18).

Future research should focus on investigating resveratrol in experimental models of pancreatic disease and in clinical populations with pancreatogenic diabetes. In particular, studies evaluating both endocrine and exocrine pancreatic outcomes–such as insulin secretion, C-peptide levels, pancreatic enzyme activity, and markers of pancreatic fibrosis–would provide valuable insight into the potential role of resveratrol in disorders involving the exocrine–endocrine pancreatic axis.

## 5 Conclusion

In conclusion, current evidence suggests that resveratrol exerts multiple beneficial effects on metabolic control and pancreatic function in diabetes mellitus. These mechanisms may also be relevant to conditions characterized by combined exocrine and endocrine pancreatic dysfunction, such as pancreatogenic diabetes, although direct clinical evidence in this population is currently lacking. By protecting acinar cells from oxidative damage through Nrf2 activation and inhibiting inflammatory cascades via NF-κB and NLRP3 suppression, resveratrol could preserve exocrine secretion, mitigate nutrient malabsorption, and prevent secondary beta-cell loss–addressing PDM’s core pathophysiology of concurrent exocrine insufficiency and endocrine dysfunction. Preclinical models demonstrate its ability to reduce pancreatic fibrosis, apoptosis, and inflammatory cell migration, while enhancing stem cell-based regeneration to repair damage from pancreatitis or surgery. In human contexts, its insulin-sensitizing effects may stabilize brittle glycemic control, countering deficiencies in insulin, glucagon, and pancreatic polypeptide.

Despite challenges like low bioavailability–necessitating optimized formulations such as micronized or liposomal versions–resveratrol’s safety profile, with minimal gastrointestinal side effects and no hepatotoxicity or nephrotoxicity, supports its potential as a safe adjunct to standard therapies like insulin and pancreatic enzyme replacement. Ultimately, targeted clinical trials in PDM populations are essential to validate these implications and harness resveratrol’s therapeutic potential for this understudied diabetes subtype.

## Data Availability

The original contributions presented in this study are included in this article/supplementary material, further inquiries can be directed to the corresponding author.
